# Impact of personal protective equipment in preventing occupational injuries: a systematic review and meta-analysis

**DOI:** 10.3389/fpubh.2025.1720363

**Published:** 2025-12-05

**Authors:** Weiner Santos, Alejandro Lorente, Carmen Rojas, Gonzalo Mariscal, Rafael Lorente

**Affiliations:** 1 International Doctoral School, University of Extremadura, Badajoz, Spain; 2 Local Health Unit Litoral Alentejano (ULSLA), Santiago do Cacém, Portugal; 3 Ankle and Foot Surgery Unit, Department of Traumatology and Orthopaedic Surgery, University Hospital Ramón y Cajal, Madrid, Spain; 4 School of Industrial Engineering, University of Extremadura, Badajoz, Spain; 5 Institute for Research on Musculoskeletal Disorders, Valencia Catholic University, Valencia, Spain; 6 Department of Orthopedic Surgery and Traumatology, University Hospital of Badajoz, Badajoz, Spain

**Keywords:** occupational injury, personal protective equipment, systematic review, workplace safety, compliance

## Abstract

**Background:**

Occupational injuries remain a significant public health concern across diverse industries, with personal protective equipment (PPE) widely advocated to mitigate risk. However, the real-world effectiveness of PPE and factors influencing its use require synthesis. Our objective is to determine whether the use of personal protective equipment (PPE) reduces the occurrence of occupational injuries among workers in high-risk industries.

**Methods:**

A comprehensive search was done to include all relevant studies published between January 2000 and June 2025 in PubMed, Scopus, Web of Science, Cochrane CENTRAL, and Embase, following the PRISMA 2020 recommendations. Studies that met the criteria included adult workers in construction, mining, manufacturing, agriculture, or related fields. They examined PPE interventions (helmets, gloves, goggles, respirators, safety shoes, high-visibility apparel, and harnesses) and reported on injuries and compliance measures. Two reviewers independently screened studies, extracted data, and assessed quality using the Joanna Briggs Institute checklist. Random-effects meta-analyses were performed in R to pool injury prevalence, PPE effectiveness (ORs), and compliance rates; heterogeneity was quantified via *I*^2^, and publication bias via funnel plots and Egger's test.

**Results:**

Eighteen cross-sectional studies (*n* = 7,612 workers) were included. A meta-analysis of 15 studies (*N* = 6,325) found that only about half of workers used personal protective equipment (PPE) (pooled prevalence = 51%, 95% CI 39–62%) with extreme heterogeneity. Industry type, rather than age or gender, explained the most variability, with use being higher in industries such as heavy industry, metalwork, and manufacturing. The main barrier was a lack of PPE (72%), followed by discomfort, poor training, and time-saving motives. Compliance was highest for basic equipment and lowest for specialized equipment. Supervision and safety training significantly reduced the risk of injury (OR = 2.04 and 1.81, respectively).

**Conclusion:**

PPE use is associated with lower odds of occupational injury, and the prevalence of occupational injuries is higher when PPE is available, properly fitted, and supported by training and supervision. However, low compliance was driven primarily by supply and ergonomic factors. Integrated strategies encompassing reliable PPE provisioning, user-centered design, comprehensive training, and organizational commitment are essential to enhance workplace safety.

## 1 Introduction

Occupational injuries are still a big problem for public health around the world, especially in areas like construction, mining, manufacturing, and agriculture (1). According to the International Labour Organization, more than 2.78 million people die at work every year because of accidents or illnesses. Each year, 374 million individuals sustain injuries at work, resulting in long-term disabilities and significant work absences (2). The economic effect is just as devastating; it costs the globe around 4% of its GDP per year. In high-risk sectors, personal protective equipment (PPE), including helmets, gloves, safety goggles, masks, protective garments, and footwear, is a crucial component of efforts to prevent injuries. People generally do not follow the guidelines, and studies do not always demonstrate the effectiveness of PPE in real-world work settings, despite its widespread advertising and use (3, 4).

PPE serves as a final line of defense against workplace hazards when engineering and administrative controls are either unfeasible or insufficient. PPE represents the least effective measure in the recognized hierarchy of controls in occupational health, following elimination, substitution, engineering, and administrative controls. The success is contingent upon effective primary hazard management, a strong organizational safety culture, and the risk perception of both workers and managers. Helmets protect against traumatic brain injuries, gloves safeguard against cuts and burns, and respirators protect people from inhaling harmful substances. In real life, however, PPE's effectiveness depends on a multitude of factors, including how well it is used, how well it is maintained, how effectively workers are trained, how well it fits, and how comfortable it is. All of these elements can change how well it protects and how well it works. Numerous primary studies and reviews have examined specific types of PPE or particular types of employment; however, a comprehensive synthesis is currently lacking that evaluates the effectiveness of PPE in protecting workers across various work environments (4–7).

Additionally, the literature lacks sufficient understanding of how various factors, including the type of industry, the demographics of the workers, or the regulations in a specific area, impact the protective value of PPE. A significant amount of research has shown that only a small percentage of workers consistently use PPE. An AI-assisted audit of construction sites revealed PPE violations in nearly a third of the monitored shifts, even in high-income areas. This shows how important organizational culture and poor oversight are (8). Hakim et al. also examined eight low- and middle-income countries and found that fewer than half of the wards had enough respirators and goggles on hand (9). The difference between policy and practice highlights the need for a quantitative summary that measures the effectiveness of PPE and identifies the factors contributing to its success or failure.

This study aims to determine whether the use of personal protective equipment (PPE) reduces the occurrence of occupational injuries among workers in high-risk industries.

## 2 Materials and methods

### 2.1 Study design

This study is a systematic review and meta-analysis that investigates the effectiveness of personal protective equipment in preventing workplace injuries in various scenarios. The methodology adheres to the PRISMA 2020 guidelines, ensuring transparency, reproducibility, and methodological rigor (10).

### 2.2 Eligibility criteria

Population (P): Workers aged 18 years and older employed in construction, mining, manufacturing, or agriculture. Intervention (I): Use of personal protective equipment (PPE), including helmets, gloves, goggles, respirators, safety shoes, high-visibility clothing, and harnesses. Comparator (C): Workers not using PPE or using it inconsistently. Outcomes (O): Primary outcome: Incidence of workplace injuries, PPE usage, and compliance. Secondary outcomes: Participation in safety training, types of PPE used, and barriers to PPE use. Study design: Eligible designs included randomized controlled trials, quasi-experimental studies, cohort studies, and cross-sectional studies reporting primary data. Reviews, editorials, case reports, and qualitative research were excluded. Other criteria: Only peer-reviewed articles published in English between January 2000 and June 2025 were considered.

### 2.3 Information sources and search strategy

A comprehensive literature search was conducted using the following electronic databases: PubMed, Scopus, Web of Science, Cochrane CENTRAL, and Embase. The search included combinations of Medical Subject Headings (MeSH) and keywords such as [(personal protective equipment OR PPE) AND (occupational injur^*^) AND (workplace safety OR helmets OR gloves OR accident prevention OR industrial safety)]. The search strategy for each database is summarized in Supplementary Table S1.

### 2.4 Study selection

All articles included were imported into EndNote X9 for duplicate removal. Two independent authors screened titles and abstracts for relevance. Full texts of potentially eligible articles were then assessed for inclusion. Discrepancies were resolved through discussion or consultation with a third author. Although randomized and cohort designs were eligible, no studies of these types met the inclusion criteria after screening; therefore, all included studies were cross-sectional in design.

### 2.5 Data extraction

A standardized data extraction form was used to collect the following variables: authors, year of publication, country, study design, sample size, occupational setting, type of PPE, outcome measures, and effect estimates (including odds ratios and incidence rates).

### 2.6 Quality assessment

Two authors independently appraised methodological quality. The Joanna Briggs Institute (JBI) Critical Appraisal Checklist for Analytical Cross-Sectional Studies was used (11). It examines eight domains: clarity of inclusion criteria, adequate description of participants and setting, validity and reliability of exposure measurement, objectivity and standardization of outcome assessment, identification of potential confounders, strategies for controlling confounding, appropriateness of statistical analysis, and sufficiency of response rate with proper handling of missing data.

### 2.7 Data synthesis and statistical analysis

Meta-analysis was conducted using R software. Random-effects models were employed due to anticipated heterogeneity. Effect sizes were calculated as odds ratios (ORs) with 95% confidence intervals (CIs). Heterogeneity was assessed using the *I*^2^ statistic. Publication bias was evaluated using funnel plots and Egger's test.

## 3 Results

### 3.1 Literature review

**Figure 1:**
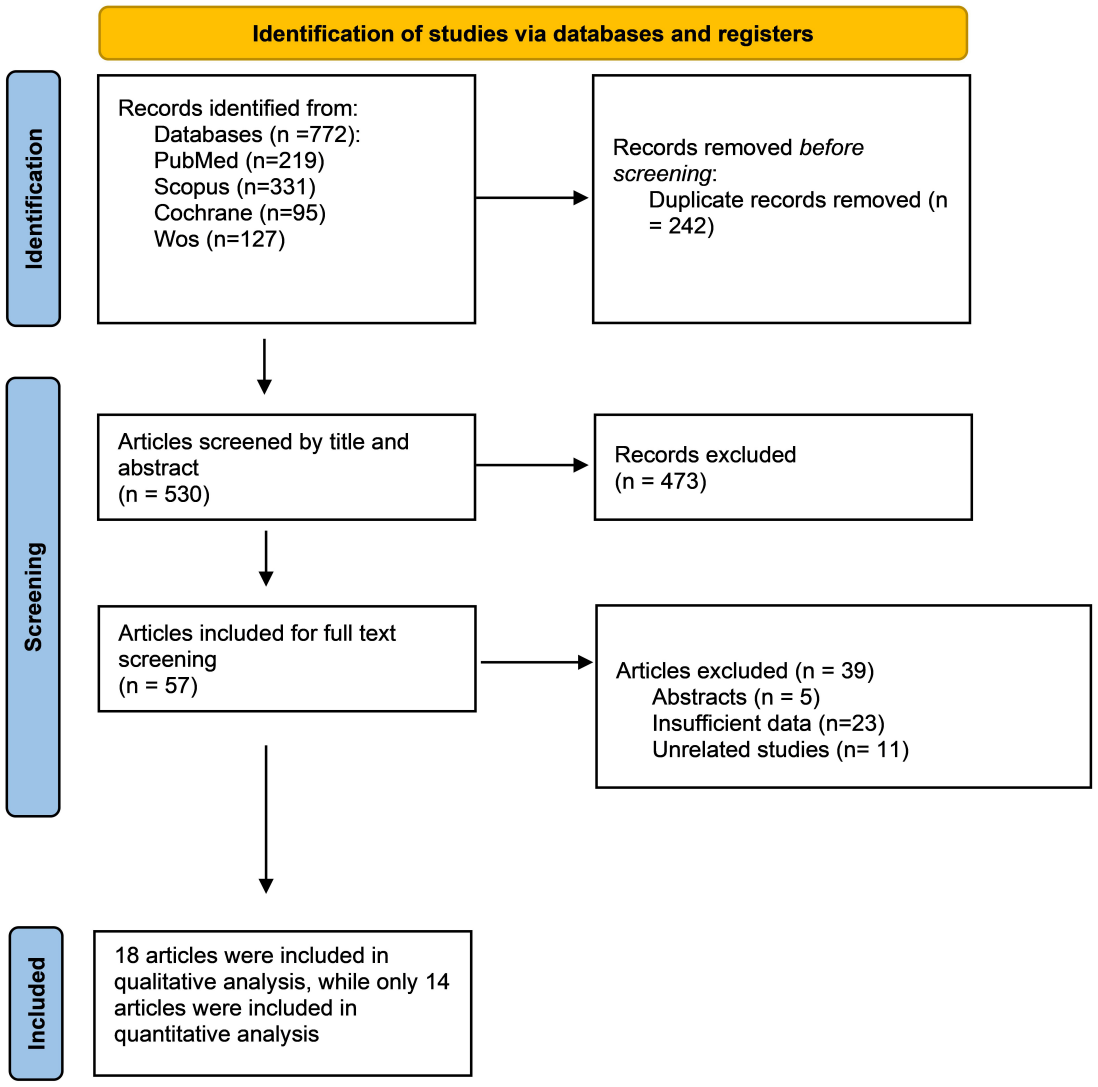
Prisma flow diagram.

A literature search yielded 772 records; after removing duplicates, 530 articles were screened based on title and abstract. Finally, 57 full texts were reviewed by two independent authors. Eighteen studies met all eligibility criteria (3, 4, 6, 7, 12–25) (Figure 1).

### 3.2 Study characteristics

All 18 included articles were cross-sectional studies and are presented in Table 1. Collectively, the studies encompassed 7,612 workers drawn from a wide variety of industrial settings, most commonly construction (eight studies), metal manufacturing (five studies), and waste collection (one study). Sample sizes ranged from 102 to 803 participants, with a median male proportion of 77%. Mean ages clustered in the mid-twenties to late thirties, reflecting a predominantly young adult workforce. Educational attainment varied markedly; in Iranian and Thai cohorts, more than half of workers had completed secondary or higher education, whereas in several Ethiopian studies, fewer than one-third had progressed beyond primary school.

**Table 1:** Baseline characteristics of included studies.

**Study (year)**	**Country**	***N* (male/female)**	**% male**	**Mean age ±SD (or range)**	**Marital status (%)**	**Education (%)**	**Industry/type of work**
Khoshakhlagh 2024	Iran	369 (346/23)	93.8	35.2 ± 12.3	Married 66.4%, Single 33.6%	Sec 35.8%, High 64.2%	Small- and medium-sized manufacturing enterprises
Kwangsukstith 2025	Thailand	709 (487/222)	68.7	NR	Married 27.1%, Single 66.1%, Div/Wid/Sepr 6.8%	Illit 4.9%, Sec 45.8%, High 49.2%	Motorcycle food-delivery riders
Balkhyour 2019	Saudi Arabia	102 (NR)	NR	34.9 (21–52)	Married 86.2%, Single 11.8%, Div/Wid/Sepr 2.0%	Illit 28.4%, Prim 11.8%, Sec 46.1%, High 13.7%	Metal workshops (expatriate workers)
Aliyi 2024	Ethiopia	393 (302/91)	76.8	24.7 (18–49)	Married 42.5%, Single 57.5%	Illiteracy is 8.4%, Primary is 46.1%, Secondary is 36.6%, and High is 8.9%.	Building-construction workers
Kilfe 2014	Ethiopia	442 (428/14)	96.8	37.7 ± 10.7	Married 62.0%, Single 30.2%, Div/Wid/Sepr 7.2%		Iron & steel industries
Megene 2020	Ethiopia	443 (432/11)	97.5	27.5 ± 6.3	44.5% of individuals are married, 50.1% are single, 3.3% are divorced, widowed, or separated, and 0.7% are widowed.	Illite: 6.5%, Primary: 34.8%, Secondary: 32.5%, High-grade: 16.7%	Iron & steel industries
Benti 2019	Ethiopia	583 (514/69)	88.2	18–30	Married 51.6%, Single 45.6%, Div/Wid/Sep 1.2%, Widowed 1.5%	Illite constitutes 2.1%, Primary minerals make up 20.2%, Secondary minerals account for 52.5%, and High minerals represent 25.2%.	Metal-manufacturing factories
Temesgen 2022	Ethiopia	351 (223/128)	63.5	38 ± 12	Married 63.8%, Single 19.9%, Div/Wid/Sepr 12.1%, Widowed 4.2%	Illit: 63.3%, Prim: 28.6%, High: 8.1%.	Municipal solid-waste collectors
Gebremeskel 2019	Ethiopia	374 (193/181)	51.6	25.7 ± 6.7	Married 34.8%, Single 65.2%	Illite constitutes 21.9%, Primary minerals make up 42.8%, Secondary minerals account for 26.5%, and High minerals represent 8.8%.	Building-construction workers
Lette 2019	Ethiopia	398 (306/92)	76.9	18–35	Married 70.6%, Single 26.9%, Div/Wid/Sep 2.5%	Illit 19.8%, Prim 58.0%, Sec 22.2%	Building-construction workers
Lette 2018	Ethiopia	355 (266/89)	74.9	26.4 ± 9.4	Married 40.6%, Single 55.8%, Div/Wid/Sep 3.1%, Widowed 0.6%	Illit 14.0%, Prim 45.6%, Sec 40.4%	Building-construction workers
Hunegnaw 2021	Ethiopia	803 (212/591)	26.4	28.9 ± 8.0	Married 47.8%, Single 45.3%, Div/Wid/Sep 5.0%, Widowed 1.9%	Illite is present at 3.0%, Primary minerals at 12.7%, Secondary minerals at 19.7%, and High minerals at 64.6%.	Mixed industrial workers
Girma 2022	Ethiopia	404 (328/76)	81.2	28 (18–50)	NR	Prim 42.3%, Sec 38.1%, High 19.6%	Small-scale woodworking
Ona 2024	Ethiopia	297 (219/78)	73.7	34.5 ± 6.1	NR	NR	Large-scale manufacturing factories
Yosef 2023	Ethiopia	368 (179/189)	48.6	27.2 ± 8.4	Married 45.9%, Single 54.1%	Illite constitutes 1.1%, Primary minerals make up 12.5%, Secondary minerals account for 20.1%, and High minerals represent 64.1%.	Industrial-park construction
Alemu 2020	Ethiopia	206 (157/49)	76.2	25.5 ± 4.1	Married 39.3%, Single 59.2%, Div/Wid/Sepr 1.5%	Illit 30.1%, Prim 26.2%, Sec 23.8%, High 19.9%	Building-construction workers
Gupta 2017	India	309 (288/21)	93.2	NR	NR	NR	Railway-wagon repair workshop
Sehsah 2020	Egypt	384 (≈ all male)	NR	NR	Married 69.8%, Single 13.8%, Div/Wid/Sepr 16.4%	Illit 42.7%, Prim 32.6%, Sec 24.7%	Construction workers

The bold values represent statistically significant results.

### 3.3 Quality assessment

Using the JBI tool, three studies achieved a high-quality assessment with a JBI rating (≥7/8), while 14 studies were of moderate quality (5–6/8), and only one study was classified as low quality (4/8). Across the checklist, we identified the most frequent weaknesses as inadequate adjustment for confounding variables (Domain 6) and insufficient reporting of response rates (Domain 8) (Supplementary Table S2).

### 3.4 Prevalence of PPE use

Fifteen studies (*N* = 6,325 workers; 3,323 PPE users) reported point estimates. A meta-analysis showed a pooled prevalence of 51% (95% CI 39–62%), indicating that roughly half of the surveyed workers reported using any PPE on the job. Heterogeneity was extreme (τ^2^ = 0.81; *I*^2^ = 98.6 %, *Q* = 985.8, *p* < 0.0001). A funnel plot was generated to assess publication bias; no significant asymmetry was detected (Supplementary Figure S1).

Meta-regression was done to explore heterogeneity. Neither the mean age (β = +0.06 on the logit scale per year, *P* = 0.25) nor the proportion of males (β = +0.38, *P* = 0.77) accounted for any appreciable heterogeneity (*R*^2^ = 0% in both models; residual *I*^2^ ≈ 99%). Furthermore, contextual factors inherent to industrial settings emerged as the primary drivers; meta-regression using sector as a factor confirmed the subgroup findings: relative to the reference category (Construction), Heavy Industry (β = +1.41, *P* = 0.006), Metal Manufacture (+1.71, *P* = 0.013), and Manufacturing (+2.06, *P* = 0.003) had significantly higher logit prevalences, whereas SMEs and Woodwork did not differ significantly. This model explained approximately 50% of the between-study variance (*R*^2^ = 49.9%), as shown in Table 2.

**Table 2:** Meta-regression for PPE usage based on age, the number of males, and the work sector of contributors.

**Moderator (per unit ↑)**	**β (log odds)**	**95% CI**	***p*-value**	**Adjusted OR (95 % CI)**
Mean age (yrs)	+0.061	−0.042 to +0.163	0.25	1.06 (0.96–1.18)
Proportion male (0 → 1)	+0.383	−2.202 to +2.968	0.77	1.47 (0.11–19.46)
**Sector**				
Construction (Intercept)	−0.43	−0.91 to +0.05	0.08	0.65 (0.40–1.05)
Heavy Industry	**+1.41**	+0.40 to +2.43	0.006	4.11 (1.49–11.39)
Manufacturing	**+2.06**	+0.69 to +3.43	0.003	7.84 (2.00–30.76)
Metal Manufacture	**+1.71**	+0.36 to +3.07	0.013	5.53 (1.43–21.44)
Mixed Industry	+0.98	−0.37 to +2.32	0.16	2.65 (0.69–10.21)
SMEs	−0.26	−1.62 to +1.10	0.71	0.77 (0.20–2.99)
Solid Waste	+0.57	−0.78 to +1.93	0.41	1.78 (0.46–6.89)
Woodwork	−0.98	−2.35 to +0.38	0.16	0.37 (0.10–1.46)

The pooled proportions of workers reporting specific reasons for non-compliance with PPE use are presented in Table 3. The most prevalent barrier was Lack of PPE/not provided (pooled proportion = 0.72, 95% CI: 0.69–0.75; *k* = 4), with no observed heterogeneity (*I*^2^ = 0%). Moderately prevalent reasons included Uncomfortable (0.27, 95% CI: 0.23–0.32; *k* = 3; *I*^2^ = 12.6%), Lack of training (0.34, 95% CI: 0.09–0.74; *k* = 3; *I*^2^ = 98.3%), and Save time (0.36, 95% CI: 0.04–0.87; *k* = 2; *I*^2^ = 98.8%).

**Table 3:** Reasons for not using PPE.

**Because**	***k***	**Pooled prop (95 % CI)**	***I* ^2^**
Lack of PPE/not provided	4	0.72 (0.69–0.75)	0%
Uncomfortable	3	0.27 (0.23–0.32)	12.6%
Save time	2	0.36 (0.04–0.87)	98.8%
Lack of training	3	0.34 (0.09–0.74)	98.3%

K refers to the number of studies.

### 3.5 PPE item compliance pattern

To explore the compliance of each tool, a systematic review was conducted by collecting relevant data, as shown in Supplementary Table S3. The highest levels of adherence were recorded in Gupta's 2017 (7) wagon-repair workshop, where regular use reached 100% for safety shoes, heavy-duty gloves, and hard hats. By contrast, only 0.4% of Iranian SME workers (15) reported wearing a helmet often or always, and harness use was reported by ≤ 2% of the same cohort. Thai food-delivery riders almost universally reported always wearing helmets (94%) and face masks (92%) (4). Conversely, only one-fifth of that group and fewer than one-third of Egyptian construction laborers consistently used goggles/safety glasses (6). Across studies, items requiring fit testing or specialist training, such as respirators, ear protection, and fall-arrest devices, consistently showed the lowest uptake.

### 3.6 Supervision

A meta-analysis of five studies examining the association between supervision and occupational injuries revealed a significantly elevated pooled odds ratio (OR = 2.04, 95% CI: 1.45–2.87; z = 4.09, *p* < 0.0001). Moderate heterogeneity was observed (*I*^2^ = 58.8%, *p* = 0.0636), as seen in Supplementary Figure S2. Sensitivity analysis was performed by excluding Benti et al.; we found that heterogeneity was reduced, with *I*^2^ equal to 19.8%, as shown in Supplementary Figure S3.

### 3.7 Safety training

**Figure 2:**
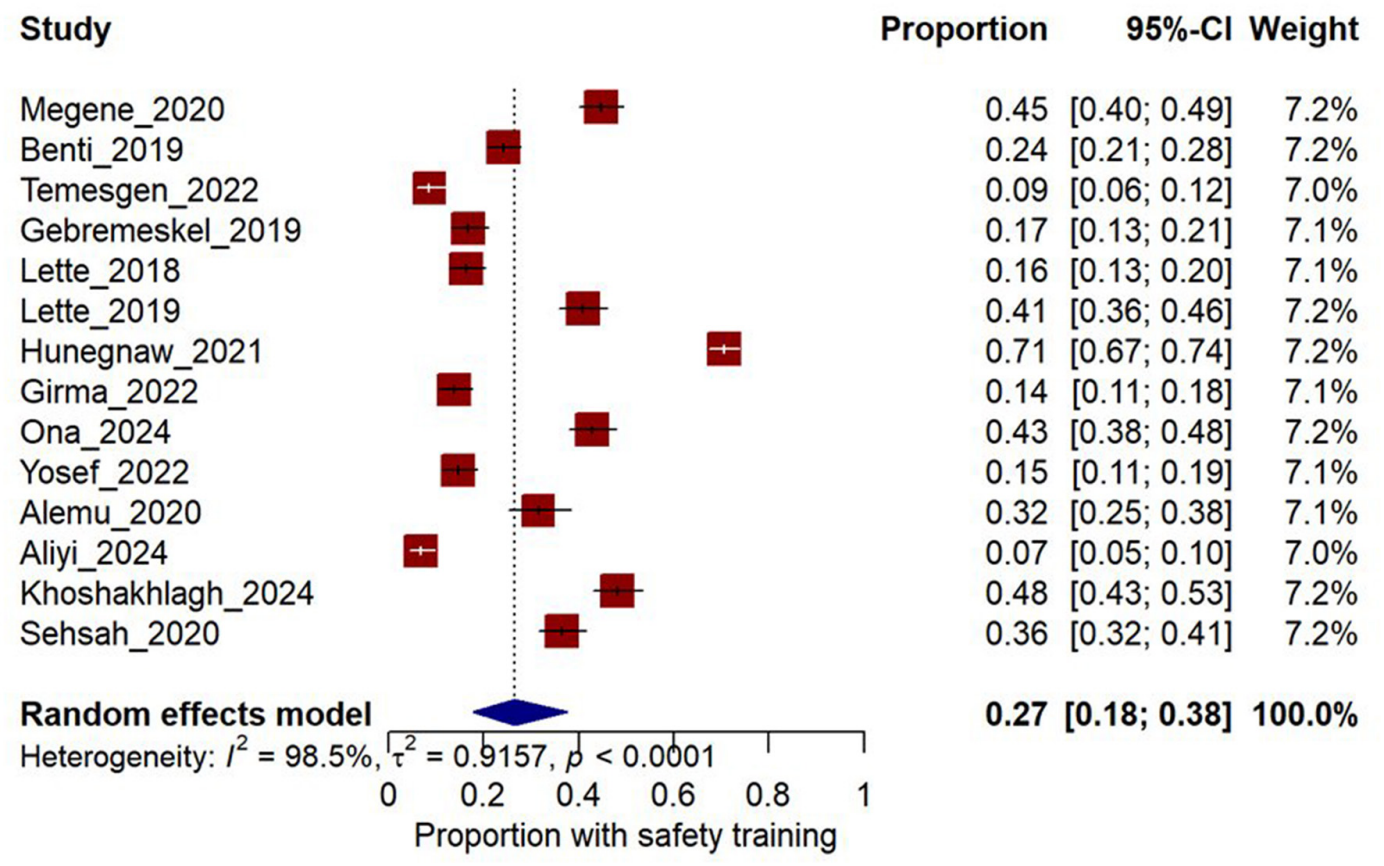
Forest plot of the pooled ratio of safety training prevalence.

Fourteen cross-sectional studies included 5,883 workers, with 1,913 of them reporting prior safety training. A meta-analysis revealed a pooled prevalence of 27% (95% CI, 18–38%). Heterogeneity was high (τ^2^ = 0.92, *I*^2^ = 98.5%, *Q* = 892.2, df = 13, *p* < 0.0001), as seen in Figure 2. To explore whether workforce demographics explained this heterogeneity, univariable meta-regressions were performed using mean age and proportion of male workers as study-level moderators. Still, neither predictor was statistically significant, as summarized in Table 4.

**Table 4:** Meta-regression for safety training.

**Moderator**	**β (logit scale)**	**SE**	***z***	***p*-value**
Mean age (years)	+0.033	0.064	0.51	0.61
% male workers	−0.284	1.400	0.20	0.84

Moreover, six studies examined the association between safety-training status and occupational injury. Our meta-analysis revealed an overall odds ratio of 1.81 (95% CI, 1.41–2.32; *z* = 4.66, *p* < 0.0001), indicating that workers without formal safety training were approximately two and a half times more likely to sustain an injury than their trained counterparts. Moderate heterogeneity was observed (*I*^2^ = 34.5%, *p* = 0.1774), as shown in Supplementary Figure S4. Sensitivity analysis was performed by excluding Benti et al.; we found that heterogeneity was reduced, with an *I*^2^ value of 0%, as shown in Supplementary Figure S4.

## 4 Discussion

Occupational injuries are one of the most important causes of death worldwide. This meta-analysis examined prior evidence indicating that PPE tools, including helmets, gloves, goggles, respirators, protective footwear, and clothing, substantially reduce the prevalence of occupational injuries across various industries. Although this review quantifies PPE-related outcomes, the observed associations may also reflect workplaces with inherently stronger occupational health cultures. Sites with effective hazard controls and supervision are also more likely to provide and enforce PPE, making it difficult to isolate PPE effects from broader safety management practices.

PPE comprises a wide variety of tools intended to safeguard specific body regions from occupational hazards, including skull protection (hard hats, bump caps), ocular and facial protection (safety glasses, goggles, face shields), auditory protection (earplugs, earmuffs), respiratory protection (filtering facepiece respirators, powered air-purifying respirators), manual and forearm protection (chemical-resistant, cut-resistant, and heavy-duty gloves), pedal and leg protection (steel-toe boots, puncture-resistant soles), bodily protection (high-visibility apparel, chemical suits, coveralls), and fall-arrest systems (harnesses, lanyards, anchor points) (3, 6, 13).

This meta-analysis found that the incidence of occupational injuries was 41%. This finding is consistent with a previous meta-analysis, which reported a 12-month prevalence of 46.8% among construction workers (26).

Hulshof et al. reported that annual injury proportions typically range from 10 to 30%.

Age and gender were not associated with PPE usage, as found in meta-regression. The non-significance of age and gender suggests that organizational and ergonomic factors, such as supervision, PPE availability, and comfort, are stronger determinants of compliance than demographic attributes. Studies in healthcare and industrial settings consistently demonstrate that demographic factors have a negligible impact on predicting PPE compliance when organizational and ergonomic variables are considered. For example, a large cross-sectional survey of sanitary workers in public hospitals found that neither age nor gender predicted PPE adherence after adjusting for training and availability of equipment (27, 28).

Our pooled compliance estimate of 51% is marginally lower than the 60% utilization reported in a 2023 African meta-analysis of industrial workers (29). Ethiopian construction sites have demonstrated that supervisory enforcement of safety rules is the strongest predictor of compliance, enhancing individual knowledge and attitudes (5).

Only one worker in four had ever received formal safety instruction (pooled prevalence, 27 %), yet the lack of training increased injury odds by 81%. Meta-reviews of OHS education concur that training alone cannot eliminate hazards but does improve proximal outcomes, knowledge, safe behavior, and, in higher-quality trials, incident rates (30).

This study also showed a high prevalence of PPE use and a significant relationship between failure to use it and occupational injuries. This agrees with previous studies in which workers who did not use PPE were found to have nearly three times the odds of sustaining an occupational injury compared to those who did.

Malta et al. identified a marked disparity in PPE use between low- and high-HDI countries, noting an overall indifference regarding whether individual safety devices were used, despite evolving regulations (31). Sehsah et al. showed a strong correlation between inconsistent PPE use and higher incident rates in construction workers (6). A meta-analysis of firefighters underscored that while PPE is protective, physiological strain from heat and weight can hinder compliance (32). Another study demonstrated that the proper use of helmets, gloves, eye protection, and respiratory devices can reduce head, hand, and facial injuries by 40–70% (33).

Recent scoping reviews show wearable sensors (accelerometers, inertial measurement units) achieve up to 95% accuracy in posture correction and fatigue monitoring, offering real-time hazard detection that can help in reducing musculoskeletal injuries by 20–30% in pilot trials (34, 35).

## 5 Limitations and implications

This review has several limitations that should be taken into consideration. First, all included studies were cross-sectional, which limits causal inference and introduces the potential for reverse causality. Moreover, heterogeneity across analyses limits the precision of pooled estimates, suggesting that there should be more standardized injury surveillance. Additionally, extreme heterogeneity is likely to stem from differences in industrial context, regulatory environments, PPE definitions, and reporting standards, rather than random variation. Furthermore, reliance on self-reported outcomes raises concerns about recall and social desirability biases. We were unable to analyze the prevalence of each PPE technology and regulatory landscape, which may mask important trends. Certain high-risk industries (e.g., oil and gas, healthcare) were underrepresented, which limited the generalizability of the findings; additionally, despite searching five major databases, relevant studies in specialized or regional repositories may have been overlooked. Nevertheless, our findings clearly indicate that enhancing PPE availability, tailoring equipment to user needs, strengthening training and supervision, and focusing on vulnerable groups should be cornerstones of occupational safety interventions. Future research should prioritize longitudinal evaluations of multifaceted safety programs and explore the impact of evolving technologies, such as wearable sensors, on real-time hazard detection and injury prevention.

## 6 Conclusion

Our meta-analysis revealed that the use of personal protective equipment significantly reduces the incidence of workplace injuries. However, the effectiveness of PPE hinges on its proper use, fit, training, and enforcement. To close the gap between the availability of PPE and its effective use, we require research from multiple fields, policy changes, and engineering enhancements. To enhance the effectiveness of PPE in preventing workplace injuries, comprehensive strategies that consider these factors must be implemented.

## Data Availability

The original contributions presented in the study are included in the article/Supplementary material, further inquiries can be directed to the corresponding authors.
