# Supplementary material for: Impact of personal protective equipment in preventing occupational injuries: a systematic review and meta-analysis
Source: Front Public Health. 2025 Dec 5;13:1720363. doi: 10.3389/fpubh.2025.1720363 (PMC12715716; doi:10.3389/fpubh.2025.1720363)
Supplement: Supplementary file 1 [file Supplementary_file_1.docx]

Figure S1: Forrest plot and funnel plot of the overall prevalence of PPE usage.

**
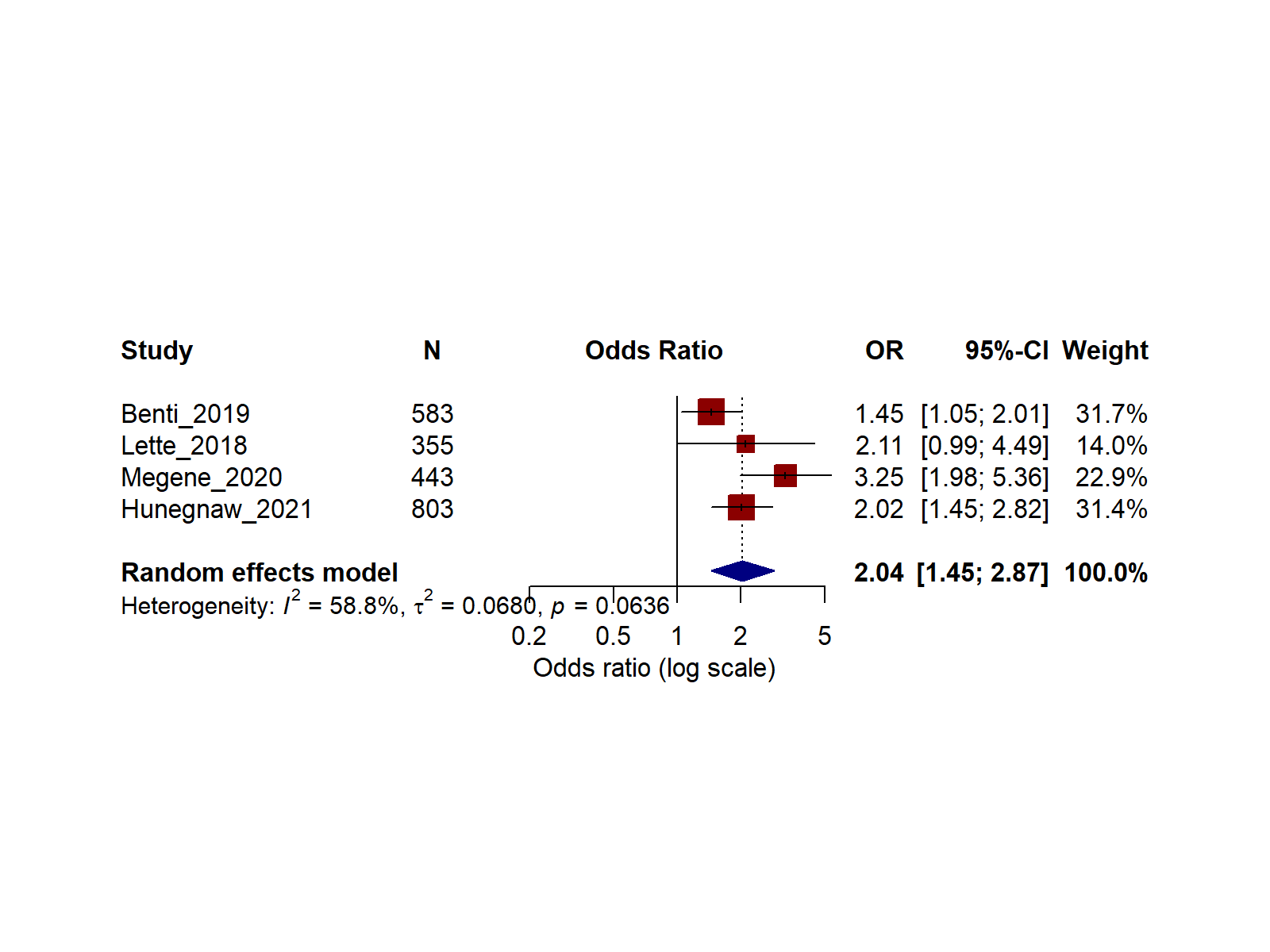
**

Figure S2: Forest plot of the pooled ratio of supervision association with occupational injuries.


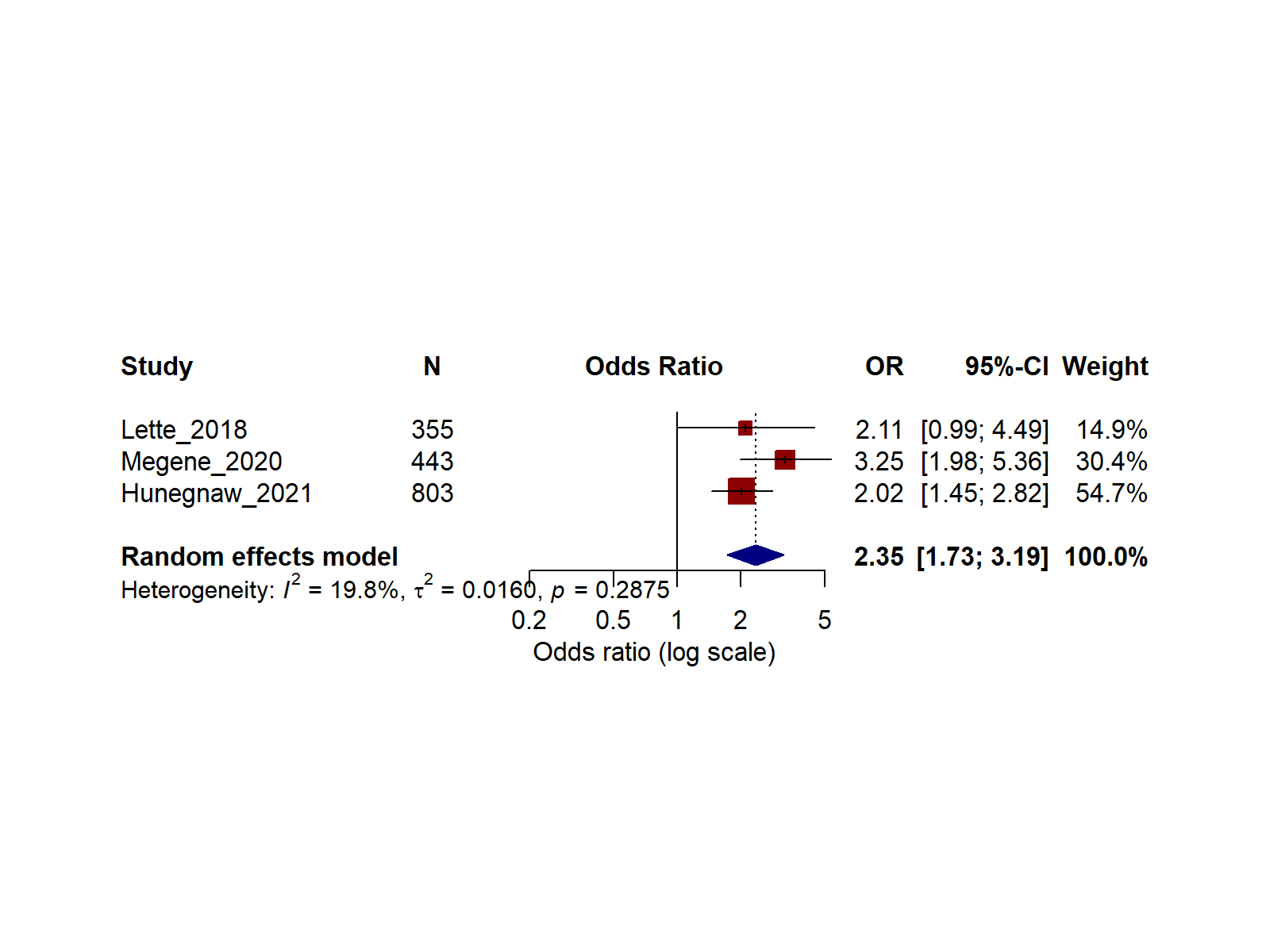


Figure S3: Forest plot of sensitivity analysis of the pooled ratio of supervision association with occupational injuries.


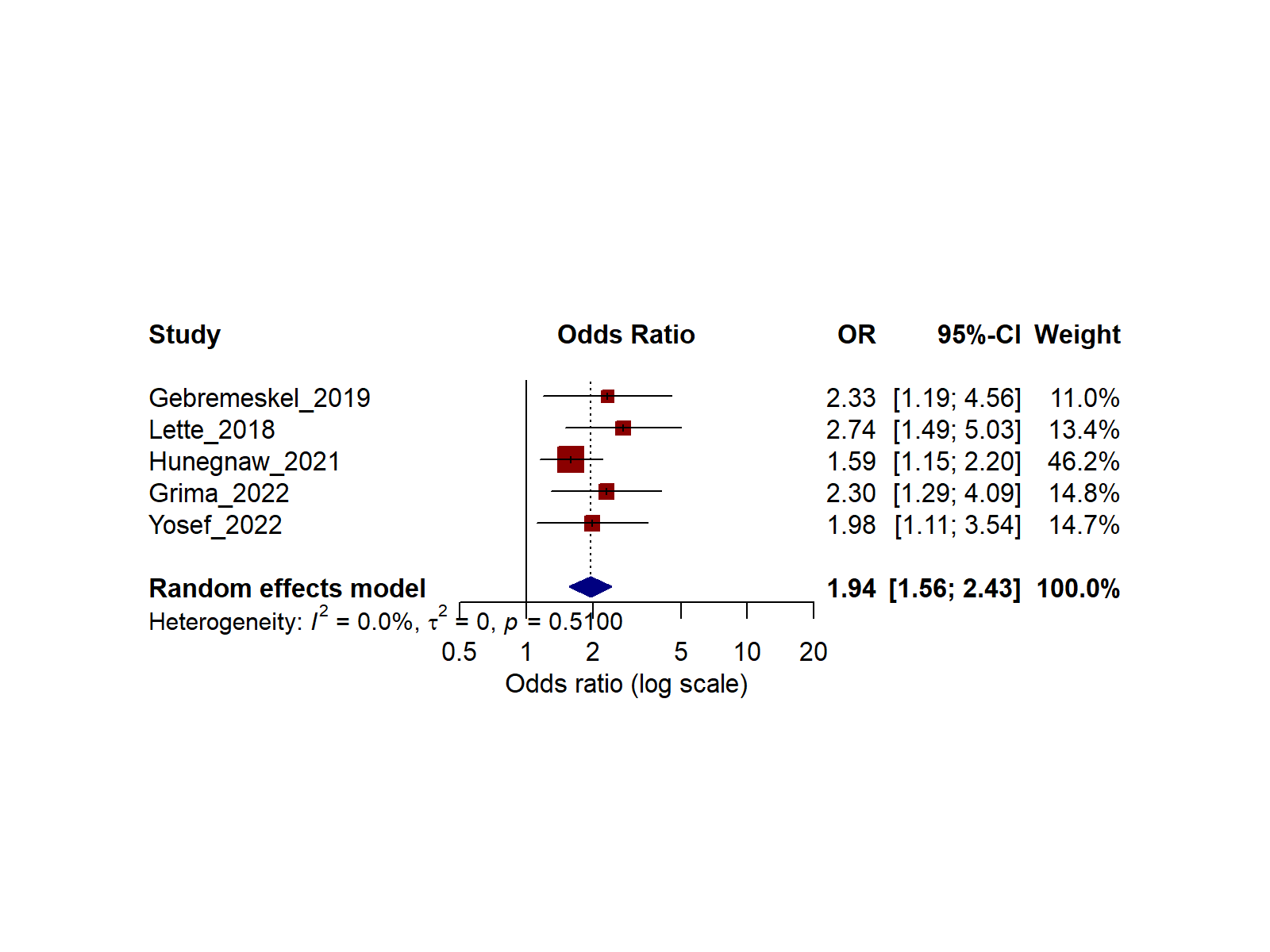


Figure S4: Forest plot of sensitivity analysis of the pooled ratio of safety training association with occupational injuries.

Table S1. Search strategy used for each electronic database.

| **Database** | **Search strategy** | **Filters and limits applied** |
| --- | --- | --- |
| **PubMed** | ("personal protective equipment"[MeSH Terms] OR "PPE" OR "safety helmet" OR "safety gloves" OR "safety goggles" OR "respirators" OR "protective footwear" OR "high-visibility clothing" OR "harness") AND ("occupational injuries"[MeSH Terms] OR "work-related injury" OR "industrial accident" OR "workplace safety" OR "occupational accident") | Filters: Humans; English; Adult (≥18 years). |
| **Scopus** | (("personal protective equipment" OR "PPE" OR "protective gear" OR "safety helmet" OR "gloves" OR "goggles" OR "respirator" OR "safety shoes" OR "protective clothing") AND ("occupational injury" OR "work-related injury" OR "industrial accident" OR "workplace safety")) | Filters: Article; English language. |
| **Web of Science Core Collection** | ("personal protective equipment" OR PPE OR "safety helmet" OR "gloves" OR "goggles" OR "respirators" OR "protective footwear" OR "high-visibility clothing" OR "harness") AND TS=("occupational injury" OR "work-related injury" OR "industrial accident" OR "workplace safety") | Filters: Document type = Article; Language = English; Year range = 2000–2025. |
| **Embase** | 'personal protective equipment'/exp OR 'ppe' OR 'safety helmet' OR 'safety gloves' OR 'safety goggles' OR 'respirator' AND ('occupational injury'/exp OR 'industrial accident' OR 'workplace safety') | Filters: Humans; English language. |
| **Cochrane CENTRAL** | ("personal protective equipment" OR PPE OR "protective clothing" OR "safety gear") AND ("occupational injury" OR "workplace accident" OR "work-related injury") | Filters: Trials and observational studies; 2000–2025; English language. |

Table S2: Quality assessment using JBI tool.

| **Study** | **D1** | **D2** | **D3** | **D4** | **D5** | **D6** | **D7** | **D8** | **Score (0–8)** | **Overall rating†** |
| --- | --- | --- | --- | --- | --- | --- | --- | --- | --- | --- |
| Khoshakhlagh 2024 | ✔︎ | ✔︎ | ✔︎ | ✔︎ | ✔︎ | ✔︎ | ✔︎ | ❍ | **7** | High |
| Kwangsukstith 2025 | ✔︎ | ✔︎ | ✔︎ | ✔︎ | ✔︎ | ❍ | ✔︎ | ❍ | **6** | Moderate |
| Balkhyour 2019 | ✔︎ | ✔︎ | ✔︎ | ✔︎ | ❍ | ❍ | ✔︎ | ❍ | **5** | Moderate |
| Aliyi 2024 | ✔︎ | ✔︎ | ✔︎ | ✔︎ | ✔︎ | ❍ | ✔︎ | ❍ | **6** | Moderate |
| Kilfe 2014 | ✔︎ | ✔︎ | ✔︎ | ✔︎ | ❍ | ❍ | ✔︎ | ❍ | **5** | Moderate |
| Megene 2020 | ✔︎ | ✔︎ | ✔︎ | ✔︎ | ✔︎ | ❍ | ✔︎ | ❍ | **6** | Moderate |
| Benti 2019 | ✔︎ | ✔︎ | ✔︎ | ✔︎ | ✔︎ | ✔︎ | ✔︎ | ❍ | **7** | High |
| Temesgen 2022 | ✔︎ | ✔︎ | ✔︎ | ✔︎ | ✔︎ | ❍ | ✔︎ | ❍ | **6** | Moderate |
| Gebremeskel 2019 | ✔︎ | ✔︎ | ✔︎ | ✔︎ | ❍ | ❍ | ✔︎ | ❍ | **5** | Moderate |
| Lette 2019 | ✔︎ | ✔︎ | ✔︎ | ✔︎ | ✔︎ | ❍ | ✔︎ | ❍ | **6** | Moderate |
| Lette 2018 | ✔︎ | ✔︎ | ✔︎ | ✔︎ | ❍ | ❍ | ✔︎ | ❍ | **5** | Moderate |
| Hunegnaw 2021 | ✔︎ | ✔︎ | ✔︎ | ✔︎ | ✔︎ | ❍ | ✔︎ | ❍ | **6** | Moderate |
| Grima 2022 | ✔︎ | ✔︎ | ✔︎ | ✔︎ | ❍ | ❍ | ✔︎ | ❍ | **5** | Moderate |
| Ona 2024 | ✔︎ | ✔︎ | ✔︎ | ✔︎ | ✔︎ | ✔︎ | ✔︎ | ❍ | **7** | High |
| Yosef 2023 | ✔︎ | ✔︎ | ✔︎ | ✔︎ | ✔︎ | ❍ | ✔︎ | ❍ | **6** | Moderate |
| Alemu 2020 | ✔︎ | ✔︎ | ✔︎ | ✔︎ | ❍ | ❍ | ✔︎ | ❍ | **5** | Moderate |
| Gupta 2017 | ✔︎ | ✔︎ | ✔︎ | ✔︎ | ✔︎ | ❍ | ✔︎ | ❍ | **6** | Moderate |
| Sehsah 2020 | ✔︎ | ✔︎ | ✔︎ | ✔︎ | ❍ | ❍ | ❍ | ❍ | **4** | Low |

**Table S3: Prevalence of PPE Usage by Item and Study Setting**

| **PPE Item** | **Study (Country, Setting)** | **Definition of "User"** | **Users (n/N)** | **Prevalence (%)** |
| --- | --- | --- | --- | --- |
| **Boots/Safety shoes** |  |  |  |  |
|  | Gupta 2017 (India, wagon-repair) | Regular | 309/309 | 100 |
|  | Kwangsukstith 2025 (Thailand, riders) | Always | 528/704 | 75 |
|  | Sehsah 2020 (Egypt, construction) | Any use | 64/228 | 28 |
|  | Khoshakhlagh 2024 (Iran, SMEs) | Often + Always | 62/267 | 23 |
|  | Balkhyour 2019 (Saudi Arabia, welders) | Yes | 11/102 | 11 |
| **Dust mask/Respirator** |  |  |  |  |
|  | Kwangsukstith 2025 (Thailand, riders) | Always face mask | 648/704 | 92 |
|  | Sehsah 2020 (Egypt, construction) | Any use | 111/228 | 49 |
|  | Balkhyour 2019 (Saudi Arabia, welders) | Yes (face mask) | 27/102 | 27 |
|  | Khoshakhlagh 2024 (Iran, SMEs) | Often + Always | 45/267 | 17 |
| **Ear protection** |  |  |  |  |
|  | Sehsah 2020 (Egypt, construction) | Any use | 80/228 | 35 |
|  | Gupta 2017 (India, wagon-repair) | Regular (plugs) | 72/309 | 23 |
|  | Khoshakhlagh 2024 (Iran, SMEs) | Often + Always | 44/267 | 17 |
|  | Gupta 2017 (India, wagon-repair) | Regular (muffs) | 33/309 | 11 |
|  | Kwangsukstith 2025 (Thailand, riders) | Always | 70/704 | 10 |
|  | Balkhyour 2019 (Saudi Arabia, welders) | Yes | 9/102 | 9 |
| **Gloves** |  |  |  |  |
|  | Gupta 2017 (India, wagon-repair) | Regular (heavy duty) | 309/309 | 100 |
|  | Kwangsukstith 2025 (Thailand, riders) | Always | 422/704 | 60 |
|  | Khoshakhlagh 2024 (Iran, SMEs) | Often + Always | 86/267 | 32 |
|  | Sehsah 2020 (Egypt, construction) | Any use (heavy duty) | 76/228 | 33 |
|  | Balkhyour 2019 (Saudi Arabia, welders) | Yes | 28/102 | 28 |
|  | Aliyi 2024 (Ethiopia, manufacturing) | Wears on duty | 14/131 | 11 |
| **Eye protection** |  |  |  |  |
|  | Gupta 2017 (India, wagon-repair) | Regular | 204/309 | 66 |
|  | Balkhyour 2019 (Saudi Arabia, welders) | Yes (safety glasses) | 34/102 | 33 |
|  | Sehsah 2020 (Egypt, construction) | Any use (goggles) | 67/228 | 29 |
|  | Kwangsukstith 2025 (Thailand, riders) | Always (sunglasses/goggles) | 141/704 | 20 |
|  | Aliyi 2024 (Ethiopia, manufacturing) | Wears on duty (glasses + mask) | 26/131 | 20 |
|  | Khoshakhlagh 2024 (Iran, SMEs) | Often + Always | 46/267 | 17 |
| **Fall protection** |  |  |  |  |
|  | Aliyi 2024 (Ethiopia, manufacturing) | Wears on duty (safety belt) | 60/131 | 46 |
|  | Sehsah 2020 (Egypt, construction) | Any use | 66/228 | 29 |
|  | Khoshakhlagh 2024 (Iran, SMEs) | Often + Always | 5/267 | 2 |
| **Head protection** |  |  |  |  |
|  | Gupta 2017 (India, wagon-repair) | Regular (hard hat) | 309/309 | 100 |
|  | Kwangsukstith 2025 (Thailand, riders) | Always | 662/704 | 94 |
|  | Gupta 2017 (India, wagon-repair) | Regular (helmet) | 234/309 | 76 |
|  | Balkhyour 2019 (Saudi Arabia, welders) | Yes (welding helmet) | 55/102 | 54 |
|  | Sehsah 2020 (Egypt, construction) | Any use | 68/228 | 30 |
|  | Aliyi 2024 (Ethiopia, manufacturing) | Wears on duty | 34/131 | 26 |
|  | Khoshakhlagh 2024 (Iran, SMEs) | Often + Always | 1/267 | 0.4 |
| **Protective clothing** |  |  |  |  |
|  | Kwangsukstith 2025 (Thailand, riders) | Always (thermal jacket) | 634/704 | 90 |
|  | Khoshakhlagh 2024 (Iran, SMEs) | Often + Always (clothing) | 141/267 | 53 |
|  | Aliyi 2024 (Ethiopia, manufacturing) | Wears on duty (proper clothes) | 35/131 | 27 |
|  | Sehsah 2020 (Egypt, construction) | Any use (overalls) | 61/228 | 27 |
